# Maternal Smoking and Metabolic Health Biomarkers in Newborns

**DOI:** 10.1371/journal.pone.0143660

**Published:** 2015-11-23

**Authors:** Fang Fang, Zhong-Cheng Luo, Anissa Dejemli, Edgard Delvin, Jun Zhang

**Affiliations:** 1 Ministry of Education-Shanghai Key Laboratory of Children’s Environmental Health, Xinhua Hospital, Shanghai Jiao-Tong University School of Medicine, Shanghai, 200092, China; 2 Departments of Obstetrics and Gynecology, Sainte-Justine Hospital Research Center, University of Montreal, Montreal, H3T 1C5, Canada; 3 Department of Clinical Biochemistry, Sainte-Justine Hospital Research Center, University of Montreal, Montreal, H3T 1C5, Canada; Universidade do Estado do Rio de Janeiro, BRAZIL

## Abstract

**Background:**

Maternal smoking has been associated with elevated risk of type 2 diabetes among the offspring in adulthood. The mechanisms underlying this fetal “programming” effect remain unclear. The present study sought to explore whether maternal smoking affects metabolic health biomarkers in fetuses/newborns.

**Methods:**

In a prospective singleton pregnancy cohort (n = 248), we compared metabolic health biomarkers in the newborns of smoking and non-smoking mothers. Outcomes included cord plasma insulin, proinsulin, insulin-like growth factor I (IGF-I), IGF-II, leptin and adiponectin concentrations, glucose-to-insulin ratio (an indicator of insulin sensitivity) and proinsulin-to-insulin ratio (an indicator of β-cell function).

**Results:**

Independent of maternal (glucose tolerance, age, ethnicity, parity, education, body mass index, alcohol use) and infant (sex, gestational age, birth weight z score, mode of delivery, cord blood glucose concentration) characteristics, the newborns of smoking mothers had lower IGF-I concentrations (mean: 6.7 vs. 8.4 nmol/L, adjusted p = 0.006), and marginally higher proinsulin-to-insulin ratios (0.94 vs. 0.72, adjusted p = 0.06) than the newborns of non-smoking mothers. Cord plasma insulin, proinsulin, IGF-II, leptin and adiponectin concentrations and glucose-to-insulin ratios were similar in the newborns of smoking and non-smoking mothers.

**Conclusions:**

Maternal smoking was associated with decreased fetal IGF-I levels, and borderline lower fetal β-cell function. Larger cohort studies are required to confirm the latter finding. The preliminary findings prompt the hypothesis that these early life metabolic changes may be involved in the impact of maternal smoking on future risk of metabolic syndrome related disorders in the offspring.

## Introduction

Cigarette smoking is a major public health problem, and constitutes a particular risk to the fetus due to the special vulnerability during developmental stage [1]. Smoking is common in pregnant women in many countries [2, 3]. An adverse intrauterine environment may have a lasting effect on chronic disease susceptibility in adulthood (Barker’s hypothesis) [4]. Epidemiological studies have demonstrated a strong association between maternal smoking and an increased risk of type 2 diabetes among the offspring in adulthood [5, 6]. Maternal smoking is a major risk factor of intrauterine growth restriction (IUGR) [7, 8] which itself is associated with an increased risk of type 2 diabetes in adulthood [9]. It is unclear whether maternal smoking affects metabolic health in utero directly leading to an increased susceptibility to type 2 diabetes in adulthood, or the effect may be solely mediated by its impact on fetal growth. So far, the mechanisms leading to elevated risk of type 2 diabetes mellitus in adulthood following *in utero* exposure to tobacco remain largely unknown. Studies in animal models suggest that smoking may affect metabolic health status in fetal or early postnatal life including impaired glucose tolerance, decreased insulin sensitivity [10], decreased pancreatic islet size and number [11]. Fetal and neonatal exposure to nicotine—a major constituent of cigarette smoke, may impair glucose homeostasis [12] and enhance the susceptibility to metabolic syndrome in rat offspring [13]. However, there is a lack of data on the impact of maternal smoking on metabolic health in human fetuses or newborns. The present study aimed to explore this question.

## Methods

### Study population

The present study was based on a prospective pregnancy cohort that was described previously [14], but addressed a new research question and did not overlap with previously reported data. Briefly, a total of 339 singleton pregnant women at three obstetric care centers in Montreal were recruited between August 2006 and December 2008. Data and specimens were collected at 24–28 and 32–35 weeks of gestation and delivery. Data on maternal and pregnancy characteristics were collected using structured study questionnaires through face-to-face interviews and medical chart reviews by trained research nurses and assistants. A total of 248 mother-infant pairs (73%) with complete data on maternal smoking and all studied cord blood biomarkers constituted the final study cohort.

### Ethics statement

The study was approved by the research ethics committee of Sainte-Justine Hospital Research Centre, University of Montreal. Written informed consent was obtained from all study participants, according to the protocol approved by the research ethics committee.

### Maternal smoking

Maternal smoking during pregnancy was based on self-reports. The women were asked to provide information on smoking at 24–28 and 32–35 weeks of gestation: did you smoke cigarette in the last three months? If the mother answered “yes” at either time point, she was defined as a smoker during pregnancy. According to this criterion, 18 were smokers (17 reported smoking at both gestational age windows) and 230 were non-smokers in the study cohort. For smokers, we further asked a question “on average, how many cigarettes you smoked per day” at both gestational age windows.

### Anthropometric measurements

Maternal pre-pregnancy BMI was calculated as self-reported weight in kilogram divided by the square of measured height in meter. Birth weight was measured to the nearest gram using the routinely available electronic weighing device in each delivery unit, and z-score was calculated based on sex- and gestational age-specific Canadian fetal growth standards [15].

### Specimens and biochemical assays

As reported previously [14], maternal blood specimens were collected at 24–28 weeks of gestation in the routine prenatal 50-g 1-h oral glucose tolerance test (OGTT) to screen for gestational diabetes mellitus. Maternal blood glucose concentrations were taken from routine prenatal clinical test records. Venous cord blood specimens were collected immediately after the delivery of the baby. All specimens collected were kept in a freezer at -80°C until assays.

Plasma glucose, insulin and proinsulin concentrations were determined by automated glucose oxidase method, automated ultrasensitive chemiluminescent immunometric assay (DXi 800, Beckman coulter, California) and quantitative enzyme-linked immuno sorbent assay (ELISA) kit, respectively. Cord plasma glucose/insulin ratio and proinsulin concentration were used as surrogate indicators of fetal insulin sensitivity, and proinsulin-to-insulin ratio as a surrogate indicator of β-cell function [14]. Leptin and total adiponectin were measured by a human leptin immunoassay kit and human adiponectin immunoassay kit, respectively [16]. Total IGF-I and total IGF-II were measured by an automated solid-phase, enzyme-labeled chemiluminescent assay (IMMULITE 2000 Siemens) and a human IGF-II ELISA kit respectively [17]. The intra-assay and inter-assay coefficients of variation of these assays were in the range of 2%-10%. The detection limits were 3.3 pmol/L for insulin, 2.0 pmol/L for proinsulin, 0.1 μg/L for leptin, 0.1 mg/L for adiponectin, 3.2 nmol/L for IGF-I and 2.0 nmol/L for IGF-II, respectively.

### Statistical analysis

All data analyses were carried out using SAS version 9.2 (SAS Institute Inc, Cary, NC). Frequencies for categorical variables and mean/ standard deviation (SD) and median for continuous variables were calculated. Chi-square, Student’s t (for maternal age, birth weight) and Wilcoxon tests (for continuous variables with skewed data distribution) were used to compare maternal and newborn’s characteristics between smokers and non-smokers. Spearman partial correlation coefficients were calculated to evaluate the associations between the number of cigarettes smoked per day and fetal metabolic health biomarkers adjusting for gestational age. Log transformation was applied to metabolic health biomarkers (positively skewed data distribution) in generalized linear models to assess the differences between smokers and non-smokers adjusting for multiple categorical and continuous covariates. The covariates included maternal glucose tolerance (50g OGTT glucose concentration), pre-pregnancy BMI, ethnicity (Caucasian: yes or no), education (university: yes or no), age (<20, 20–34, > = 35 y), parity (primiparous: yes or no), alcohol use (yes or no), infant sex, birth weight (z score), gestational age (weeks), mode of delivery (caesarean or vaginal) and cord blood glucose concentration. A two-tailed probability (*p*) value of <0.05 was considered statistically significant.

## Results

### Maternal and newborn’s characteristics

Comparing maternal smokers vs. non-smokers, there were no significant differences in pre-pregnancy BMI, age, parity, and newborn’s sex, mode of delivery and gestational age (**Table 1).** Smokers were more likely to be a Caucasian and to drink alcohol, but less likely to have completed university education. The difference in birth weight was on the borderline of statistical significance (mean: 3253 vs. 3459 g in the newborns of smokers vs. non-smokers, p = *0*.*057*). Birth weight z scores were lower in smokers but the difference was not statistically significant (smoker -0.10 vs. non-smoker 0.16, p = 0.255). There were no significant differences in maternal OGTT and cord blood glucose levels in the neonates of smokers vs. non-smokers.

**Table 1:** Maternal and newborn’s characteristics in smoking vs. non-smoking singleton pregnancies.

	Smokers	Non-Smokers	P *
	(n = 18)	(n = 230)	
**Mothers**			
50 g OGTT glucose (mg/dl)	114.2±21.4	118.6±30.8	0.70
mmol/l	6.4±1.2	6.6±1.7	0.70
Pre-pregnancy BMI	22.7±5.1	23.97±4.97	0.11
Ethnicity, Caucasian (n, %)	16 (88.9)	136 (59.1)	***0*.*01***
Age (year)	31.3±4.7	31.0±4.7	0.79
University education (n, %)	4(22.2)	123(53.5)	***0*.*02***
Primiparous (n, %)	5 (27.8)	95 (41.3)	0.38
Alcohol use (n, %)	8 (44.4)	26 (11.3)	***<0*.*01***
Gestational diabetes (n, %)	1 (5.6)	25 (10.9)	0.70
**Newborns**			
Caesarean section (n, %)	4 (22.2)	66 (28.7)	0.75
Sex, male (n, %)	10 (55.6)	123 (53.5)	1.00
Gestational age (weeks)	38.4±1.7	39.0±1.5	0.16
Birth weight (g)	3253±440	3459±442	*0*.*06*
Birth weight (z score)	-0.1±0.91	0.2±0.9	0.26
Cord blood glucose (mg/dl)	87.8±13.8	83.2±17.0	0.23
mmol/l	4.9±0.8	4.6±0.9	0.23

Data presented are Mean±SD or n (%). BMI = body mass index; OGTT = oral glucose tolerance test.

*Crude P values in Wilcoxon or t tests (where appropriate) for differences in continuous variables (mean or median), or Chi square tests for differences in categorical variables (proportion) comparing smoking vs. non-smoking pregnancies.

Among the 18 smokers, based on self-reports at 24–28 weeks and 32–35 weeks of gestation, the median (interquartile range) number of cigarettes smoked per day during mid and late gestation was 5 (1–8). There were 8 women smoked 1–4 cigarettes/day, 6 women smoked 5–9 cigarettes/day, and 4 women smoked 10–15 cigarettes/day.

### Fetal metabolic health biomarkers

Partial correlations adjusting for gestational age at birth showed that the average number of self-reported cigarettes smoked per day during mid and late gestation was positively correlated to cord plasma glucose-to-insulin ratio (r = 0.154, p = 0.017), proinsulin-to-insulin ratio (r = 0.152, p = 0.018), and negatively correlated to cord plasma insulin (r = -0.131, p = 0.041) and IGF-1 (r = -0.157, p = 0.014) concentrations, but not correlated to proinsulin, IGF-II, leptin and adiponectin concentrations (all p>0.5). The average number of cigarettes smoked per day reported at 24–28 weeks and 32–35 weeks of gestation had similar correlations with fetal metabolic health biomarkers (data not shown).

The crude comparisons showed that the neonates of smoking mothers had higher cord blood glucose-to-insulin ratios (mean: 34.9 vs. 24.4, p = 0.038), lower IGF-I concentrations (mean: 6.7 vs. 8.4 pmol/L, p = 0.05) and marginally higher proinsulin-to-insulin ratios (mean: 0.94 vs.0.72, p = 0.06) than the neonates of smoking mothers (**Table 2)**. After the adjustments for maternal (glucose tolerance, pre-pregnancy BMI, ethnicity, educational, age, parity, alcohol use) and newborn’s (sex, gestational age, birth weight z score, mode of delivery and cord blood glucose concentration) characteristics, the difference in glucose-to-insulin ratio between smokers and non-smokers became non-significant (adjusted p = 0.14), the difference in IGF-I concentrations became more statistically significant (adjusted p = 0.006), while the difference in proinsulin-to-insulin ratio was on the borderline of statistical significance (adjusted P = 0.06). There were no significant differences (crude or adjusted) in cord blood insulin, proinsulin, IGF-II, leptin and adiponectin concentrations between the newborns of smokers and non-smokers.

**Table 2:** Maternal (24–28 weeks’ gestation, 50 g OGTT blood) and cord plasma metabolic health biomarkers in singleton newborns of smoking vs. non-smoking mothers.

		Smokers	Non-Smokers		Crude	Adjusted
Biomarker	Median	Mean±SD	Median	Mean±SD	P ^a^	P ^b^
**Maternal plasma**						
Insulin, U/L	49.2	55.8±42.1	48.5	65.7±60.6	0.67	0.11
pmol/L	295.3	334.8±252.5	290.8	394.4±363.6	0.67	0.11
Proinsulin (pmol/L)	17.8	20.0±13.0	18.3	22.3±14.9	0.56	0.31
IGF-I (nmol/L)	22.1	24.4±11.0	25.0	27.5±11.2	0.21	0.35
IGF-II (nmol/L)	113.6	112.7±15.2	117.9	119.1±17.9	0.13	**0.02**
Leptin (μg/L)	60.2	80.2±59.8	79.3	84.3±47.6	0.51	0.36
Adiponectin (mg/L)	8.1	7.8±2.3	8.3	8.5±3.2	0.52	0.26
**Cord plasma**						
Insulin, U/L	3.4	4.2±3.6	4.8	6.3±6.5	0.09	0.15
pmol/L	20.4	25.3±21.7	28.6	37.8±38.7	0.09	0.15
Proinsulin (pmol/L)	12.9	18.9±16. 6	13.8	17.8±13.5	0.66	0.57
Glucose(mg/dl)-to	28.8	34.9±27.9	16.8	24.4±20. 9	**0.04**	0.14
-insulin(U/L) ratio						
Proinsulin-to-insulin ratio	0.64	0.94±0.72	0.54	0.72±0.89	*0*.*06*	*0*.*06*
IGF-I (nmol/L)	6.0	6.7±3.2	7.8	8.4±5.0	**0.05**	**0.006**
IGF-II (nmol/L)	66.2	71.9±22.4	66.2	67.3±15.2	0.57	0.48
Leptin (μg/L)	11.0	42.8±51.9	25.7	39.6±41.9	0.48	0.74
Adiponectin (mg/L)	21.9	20.0±8.1	19.8	20.8±7. 9	0.84	0.91

^a^ Crude P values comparing the neonates of smokers vs. non-smokers in non-parametric Wilcoxon test.

^b^ P values comparing smokers vs. non-smokers in log-transformed biomarker data adjusted for maternal glucose tolerance, pre-pregnancy BMI, ethnicity, age, education, parity, alcohol use, and for cord blood biomarkers further adjusted for newborn’s sex, gestational age, birth weight (z score), mode of delivery and cord blood glucose concentration using generalized linear models.

### Maternal metabolic health biomarkers

Comparing smokers vs. non-smokers, there were no significant differences in maternal plasma insulin, proinsulin, IGF-I, leptin and adiponectin concentrations in maternal 50 g OGTT blood at 24–28 weeks’ gestation (**Table 2**). Surprisingly, there were significantly lower maternal plasma IGF-II concentrations in smokers after the adjustment for glucose tolerance, pre-pregnancy BMI, ethnicity, age, education, parity and alcohol use.

## Discussion

### Main findings

To our knowledge, this is the first study exploring the possible impact of maternal smoking on fetal β-cell function in humans. Our data provide important preliminary evidence indicating that maternal smoking may decrease fetal IGF-I levels, and may also impair β-cell function, although the latter association is on the borderline of statistical significance and requires confirmation in larger studies. The new findings prompt the hypothesis that these effects in early life may be a mechanism in “programming” the long-term impact of maternal smoking on metabolic health in the offspring.

### Data interpretation

#### IGF-I and IGF-II

IGF-I is synthesized largely in the liver but also in other tissues. Classically, IGF-I is a key driver of fetal growth [18]. However, studies in mouse models and cultured insulinoma cells suggest that IGF-I also plays an important role in maintaining β-cell function by mediating IRS-2/PI3K/Akt pathway [19,20]. There have been inconsistent findings concerning whether IGF-I levels are altered in the newborns of smoking mothers, while there is a scarcity of data on IGF-II levels. We observed that cord blood plasma IGF-I but not IGF-II levels were decreased in the newborns of smoking mothers independent of fetal growth (birth weight z score). Consistent with our findings, Chelchowska and colleagues [21] studied 50 healthy pregnant women and found that cord blood IGF-I concentrations were lower (by 20%) in the newborns of smoking mothers. However, their analyses did not adjust for fetal growth. Similar to our findings, Pringle et al [22] observed that cord plasma IGF-I concentrations were lower in the babies of mothers who smoked, but IGF-II concentrations were unaffected by smoking status. In contrast, Ermis et al [23] analyzed 44 mother-infant (7-days old) pairs and observed no significant difference in IGF-I concentrations in infant serum samples according to maternal smoking status. The reasons could be due to small sample size and difference in the timing of IGF-I detection (at birth vs. after birth). Insulin is the primary driver of fetal IGF-I production [24]. We observed lower cord plasma insulin levels in the newborns of smoking mothers, but the difference did not reach statistical significance. Similarly, lower cord blood insulin and IGF-I levels have been reported in the newborns of smoking mothers [25].

Unexpectedly, we observed lower maternal IGF-II levels in smokers after the adjustment for other maternal characteristics. The mechanisms are unclear, and this finding requires confirmation in larger cohort studies.

#### β-cell function

Smoking appears to affect glucose regulation and has been associated with increased insulin resistance and altered glucose homeostasis in adults [26]. The mechanisms responsible for smoking-induced glucose dysregulation are unclear. Animal studies have demonstrated that exposure to smoking or nicotine during the prenatal and neonatal periods could impair insulin sensitivity, decrease beta cell function, impair glucose tolerance, and increase the susceptibility to metabolic syndrome in the offspring [10–13]. A recent study suggests that nicotine may increase insulin resistance through selectively activate AMP-activated protein kinase α2 (AMPKα2) in adipocytes [27]. Little is known about the relationship between smoking during pregnancy and fetal β-cell function in humans. Our study is the first to explore this relationship in humans. We observed a borderline significantly higher cord proinsulin-to-insulin ratio (adjusted p = 0.06) in the newborns of smoking vs. non-smoking mothers independent of birth weight z score and gestational age, suggesting that tobacco may have an adverse impact on β-cell function in human fetuses/newborns. This finding is supported by data from an animal model study—prenatal nicotine exposure may decrease pancreatic islet size and number (which is a surrogate for β-cell function) in rats at the 7^th^ postnatal day of life [11].

#### Leptin and adiponectin

Adiponectin and leptin are important adipose tissue secreted hormones (adipokines) regulating energy balance and insulin sensitivity [28]. A few studies have explored the effect of maternal smoking on fetal leptin and adiponectin levels, but the findings have been inconsistent. Ozkan’s and Christos’s studies reported lower serum leptin levels comparing the neonates of smoking vs. nonsmoking mothers in cord blood or venous blood at the 7^th^ postnatal day of life [29, 30]. In contrast, Helland’s and Chelchowska’s studies found no difference in cord blood leptin concentrations between the neonates of smokers and non-smokers [31, 32]. The reasons behind these inconsistencies may be partly due to the difference in sample size, the timing (early, mid or late gestation?) and method (self-report? cotinine?) in defining the smoking status. To our knowledge, only one study analyzed the effect of maternal smoking on fetal adiponectin levels. In a study of 85 healthy women, Chelchowska and colleagues observed significantly lower cord serum total adiponectin concentrations in the newborns of smoking women [32]. However, this finding could not be confirmed in our study of 238 mother-baby pairs in healthy pregnancies free of major chronic illnesses.

### Strengths and limitations

Strengths of the study are the prospective pregnancy cohort, timely collection and processing of blood specimens and high-quality biochemical assays (low intra- and inter-assay coefficients of variation). The main limitation is the relatively small number of women in the smoking group. The study was powered to detect relatively large differences, but not powered to detect moderate differences in fetal metabolic health biomarkers. Another limitation is that smoking status was based on self-reports at mid and late gestation. We could not identify women who were previous smokers and quitted smoking in early pregnancy or before pregnancy. Such quitters might be common for woman smokers in planning pregnancy or on knowledge of pregnancy. This might explain the low prevalence of current smokers (at mid and late gestation) in the study cohort. These smoking quitters would have been mixed into the non-smoker group. However, such misclassifications would only tend to decrease the observed impact of smoking on cord blood metabolic health biomarkers. Smoking has commonly been assessed through self-report or a biomarker–mostly commonly, plasma or urine cotinine (a nicotine metabolite). Although plasma or urine cotinine measurements are more objective, they only reflect smoking exposure status in a relative short time period (about three days) [33]. Self-report is efficient and uniquely captures information about historical patterns of smoking [34]. In our study, maternal smoking status was identified through interviews twice during pregnancy, and virtually all (17/18) smokers reported smoking at both 24–28 and 32–35 weeks of gestation, indicating they were persistent smokers. Also, smoking may be prone to under-reporting due to social pressure [35]. However, such under-reporting misclassifications in smoking status would most likely tend to decrease the observed differences in cord blood biomarkers between the newborns of smokers vs. non-smokers.

## Conclusions

Increasing evidence suggests that the intrauterine environment may affect fetal metabolic health [14,32,36]. Our study provides important preliminary evidence suggesting that maternal smoking may decrease fetal IGF-I levels, and may have an adverse impact on fetal β-cell function. The latter finding needs to be confirmed in larger cohort studies. We speculate that these changes in early life may be involved in the impact of maternal smoking on future risk of metabolic syndrome related disorders in the offspring.

## Supporting Information

S1 Checklist STROBE Statement—Checklist of items that should be included in reports of cohort studies. (DOC)

S2 Checklist PLOS ONE Clinical Studies Checklist. (DOCX)

## References

[1] Rogers JM (2008) Tobacco and pregnancy: Overview of exposures and effects. Birth Defects Res C Embryo Today 84:1–15. 10.1002/bdrc.20119 18383133

[2] Mathews TJ (2001) Smoking during pregnancy in the 1990s. Natl Vital Stat Rep;49:1–14. 11561426

[3] Russell T, Crawford M, Woodby L (2004) Measurements for active cigarette smoke exposure in prevalence and cessation studies: Why simply asking pregnant women isn't enough. Nicotine Tob Res 6 Suppl 2:S141–S151. 15203817 10.1080/14622200410001669141

[4] Barker DJP (1997) Fetal nutrition and cardiovascular disease in later life. Br Med Bull. 53:96–108. 9158287 10.1093/oxfordjournals.bmb.a011609

[5] Jaddoe VW, de Jonge LL, van Dam RM, Willett WC, Harris H, Stampfer MJ, et al (2014) Fetal exposure to parental smoking and the risk of type 2 diabetes in adult women. Diabetes Care 37:2966–73. 10.2337/dc13-1679 25092685

[6] La Merrill MA, Cirillo PM, Krigbaum NY, Cohn BA (2015) The impact of prenatal parental tobacco smoking on risk of diabetes mellitus in middle-aged women. J Dev Orig Health Dis 6:242–9. 10.1017/S2040174415000045 25665487 PMC6996969

[7] Power C, Jefferis BJ (2002) Fetal environment and subsequent obesity: A study of maternal smoking. Int J Epidemiol31:413–419. 11980805

[8] Vardavas CI, Chatzi L, Patelarou E, Plana E, Sarri K, Kafatos A, et al (2010) Smoking and smoking cessation during early pregnancy and its effect on adverse pregnancy outcomes and fetal growth. Eur J Pediatr 169:741–748. 10.1007/s00431-009-1107-9 19953266

[9] Whincup PH, Kaye SJ, Owen CG, Huxley R, Cook DG, Anazawa S, et al (2008) Birth weight and risk of type 2 diabetes: A systematic review. JAMA 300:2886–2897. 10.1001/jama.2008.886 19109117

[10] Chen H, Iglesias MA, Caruso V, Morris MJ (2011) Maternal cigarette smoke exposure contributes to glucose intolerance and decreased brain insulin action in mice offspring independent of maternal diet. PloS One 6:e27260 10.1371/journal.pone.0027260 22076142 PMC3208635

[11] Somm E, Schwitzgebel VM, Vauthay DM, Camm EJ, Chen CY, Giacobino JP, et al (2008) Prenatal nicotine exposure alters early pancreatic islet and adipose tissue development with consequences on the control of body weight and glucose metabolism later in life. Endocrinology 149:6289–6299. 10.1210/en.2008-0361 18687784

[12] Bruin JE, Kellenberger LD, Gerstein HC, Morrison KM, Holloway AC (2007) Fetal and neonatal nicotine exposure and postnatal glucose homeostasis: identifying critical windows of exposure. J Endocrinol 194:171–178. 17592031 10.1677/JOE-07-0050

[13] Xu D, Xia LP, Shen L, Lei YY, Liu L, Zhang L, et al (2013). Prenatal nicotine exposure enhances the susceptibility to metabolic syndrome in adult offspring rats fed high-fat diet via alteration of HPA axis-associated neuroendocrine metabolic programming. Acta Pharmacol Sin 34:1526–1534. 10.1038/aps.2013.171 24270239 PMC4002571

[14] Luo ZC, Delvin E, Fraser WD, Audibert F, Deal CI, Julien P, et al (2010) Maternal glucose tolerance in pregnancy affects fetal insulin sensitivity. Diabetes Care 33:2055–2061. 10.2337/dc10-0819 20573751 PMC2928362

[15] Kramer MS, Platt RW, Wen SW, Joseph KS, Allen A, Abrahamowicz M, et al (2001) A new and improved population-based canadian reference for birth weight for gestational age. Pediatrics 108:e35–e35. 11483845 10.1542/peds.108.2.e35

[16] Luo Z-C, Nuyt A-M, Delvin E, Fraser WD, Julien P, Audibert F, et al (2013) Maternal and fetal leptin, adiponectin levels and associations with fetal insulin sensitivity. Obesity 21:210–216. 10.1002/oby.20250 23505188

[17] Luo ZC, Nuyt AM, Delvin E, Audibert F, Girard I, Shatenstein B, et al (2012) Maternal and fetal IGF-I and IGF-II levels, fetal growth, and gestational diabetes. J Clin Endocrinol Metab 97:1720–1728. 10.1210/jc.2011-3296 22419731

[18] Reece EA, Wiznitzer A, Le E, Homko CJ, Behrman H, Spencer EM (1994) The relation between human fetal growth and fetal blood levels of insulin-like growth factors i and ii, their binding proteins, and receptors. Obstet Gynecol 84:88–95. 7516515

[19] Burks DJ, White MF (2001) Irs proteins and beta-cell function. Diabetes 50 Suppl 1:S140–S145. 11272176 10.2337/diabetes.50.2007.s140

[20] Elghazi L, Bernal-Mizrachi E (2009) Akt and pten: B-cell mass and pancreas plasticity. Trends Endocrinol Metab 20:243–251. 10.1016/j.tem.2009.03.002 19541499 PMC4456182

[21] Chelchowska M, Gajewska J, Ambroszkiewicz J, Lewandowski L, Maciejewski TM, Oltarzewski M, et al (2013) The effect of tobacco smoking on serum concentration of selected angiogenic factors and somatomedin c in pregnant women and umbilical cord blood. Przegl Lek 70:800–804. 24501799

[22] Pringle PJ, Geary MP, Rodeck CH, Kingdom JC, Kayamba-Kay's S, Hindmarsh PC (2005) The influence of cigarette smoking on antenatal growth, birth size, and the insulin-like growth factor axis. J Clin Endocrinol Metab 90:2556–2262. 15713720 10.1210/jc.2004-1674

[23] Ermis B, Altinkaynak K, Yildirim A, Ozkan B (2004) Influence of smoking on serum and milk of mothers, and their infants' serum insulin-like growth factor-i and insulin-like growth factor binding protein-3 levels. Horm Res 62:288–292. 15542930 10.1159/000081974

[24] Gluckman PD, Pinal CS (2003) Regulation of fetal growth by the somatotrophic axis. J Nutr 133:1741S–1746S. 12730493 10.1093/jn/133.5.1741S

[25] Ingvarsson RF, Bjarnason AO, Dagbjartsson A, Hardardottir H, Haraldsson A, Thorkelsson T (2007) The effects of smoking in pregnancy on factors influencing fetal growth. Acta Paediatr 96:383–386. 17407461 10.1111/j.1651-2227.2007.00103.x

[26] Sargeant LA, Khaw K-T, Bingham S, Day NE, Luben RN, Oakes S, et al (2001) Cigarette smoking and glycaemia: The epic-norfolk study. Int J Epidemiol 30:547–554. 11416081 10.1093/ije/30.3.547

[27] Wu Y, Song P, Zhang W, Liu J, Dai X, Liu Z, et al (2015) Activation of AMPKα2 in adipocytes is essential for nicotine-induced insulin resistance in vivo. Nat Med 21:373–382. 10.1038/nm.3826 25799226 PMC4390501

[28] Zhang H, Zhang C (2010) Adipose “talks” to distant organs to regulate insulin sensitivity and vascular function. Obesity 18:2071–2076. 10.1038/oby.2010.91 20395945 PMC2946982

[29] Ozkan B, Ermis B, Tastekin A, Doneray H, Yildirim A, Ors R (2005) Effect of smoking on neonatal and maternal serum and breast milk leptin levels. Endocr Res 31:177–183. 16392620 10.1080/07435800500371748

[30] Mantzoros CS, Varvarigou A, Kaklamani VG, Beratis NG, Flier JS (1997) Effect of birth weight and maternal smoking on cord blood leptin concentrations of full-term and preterm newborns. J Clin Endocrinol Metab 82:2856–2861. 9284710 10.1210/jcem.82.9.4248

[31] Helland IB, Reseland JE, Saugstad OD, Drevon CA (2001) Smoking related to plasma leptin concentration in pregnant women and their newborn infants. Acta Paediatr 90:282–287. 11332168

[32] Chelchowska M, Ambroszkiewicz J, Mazur J, Lewandowski L, Maciejewski TM, Oltarzewski M, et al (2014) Effect of tobacco smoking on the maternal and fetal adipokine axis in relation to newborn birth weight and length. Przegl Lek 71:567–71. 25799845

[33] Dempsey D, Jacob P, Benowitz NL (2002) Accelerated metabolism of nicotine and cotinine in pregnant smokers. J Pharmacol Exp Ther 301:594–598. 11961061 10.1124/jpet.301.2.594

[34] Dukic VM, Niessner M, Pickett KE, Benowitz NL, Wakschlag LS (2009) Calibrating self-reported measures of maternal smoking in pregnancy via bioassays using a monte carlo approach. Int J Environ Res Public Health 6:1744–1759. 10.3390/ijerph6061744 19578458 PMC2705215

[35] Pickett KE, Kasza K, Biesecker G, Wright RJ, Wakschlag LS (2009) Women who remember, women who do not: A methodological study of maternal recall of smoking in pregnancy. Nicotine Tob Res 11:1166–1174. 10.1093/ntr/ntp117 19640836 PMC2746835

[36] Zhao JP, Levy E, Fraser WD, Julien P, Delvin E, Montoudis A, et al (2014) Circulating docosahexaenoic acid levels are associated with fetal insulin sensitivity. PLoS One. 9:e85054 10.1371/journal.pone.0085054 24454790 PMC3890289

